# Parental Involvement in the Transition from Paediatric to Adult Care for Youth with Chronic Illness: A Scoping Review of the North American Literature

**DOI:** 10.1155/2023/9392040

**Published:** 2023-11-24

**Authors:** Bryn Badour, Amanda Bull, Abha A. Gupta, Raza M. Mirza, Christopher A. Klinger

**Affiliations:** ^1^Faculty of Arts and Science: Health Studies Program, University of Toronto, Toronto, Ontario, Canada M5S 3G3; ^2^National Initiative for the Care of the Elderly (NICE), Toronto, Ontario, Canada M5S 1V4; ^3^Temerty Faculty of Medicine: Department of Paediatrics, University of Toronto, Toronto, Ontario, Canada M5S 1A8; ^4^Department of Paediatrics, The Hospital for Sick Children, Toronto, Ontario, Canada M5G 1X8; ^5^Division of Medical Oncology, University Health Network: Princess Margaret Cancer Centre, Toronto, Ontario, Canada M5G 2C1; ^6^Temerty Faculty of Medicine: Translational Research Program, University of Toronto, Toronto, Ontario, Canada M5S 1A8; ^7^Factor-Inwentash Faculty of Social Work: Institute for Life Course and Aging, University of Toronto, Toronto, Ontario, Canada M5S 1V4

## Abstract

With medical advancements and improvements in medical technology, an increasing number of children with chronic conditions survive into adulthood. There is accordant growing interest toward supporting adolescents throughout the transition from paediatric to adult care. However, there is currently a paucity of research focusing on the role that these patients' parents should play during and after the transition to adult care and if maintained parental involvement is beneficial during this transition within a North American context. Accordingly, this scoping review utilized Arksey and O'Malley's five-step framework to consider parental roles during chronically ill children's transition to adult care. APA PsycInfo, CINAHL, EMBASE, MEDLINE, ProQuest, and Scopus were searched alongside advanced Google searches. Thematic content analysis was conducted on 30 articles meeting the following inclusion criteria: (1) published in English between 2010 and 2022, (2) conducted in Canada or the United States, (3) considered adolescents with chronic conditions transitioning to adult care, (4) family being noted in the title or abstract, and (5) patient populations of study not being defined by delays in cognitive development, nor mental illness. Three themes emerged from the literature: the impacts of maintaining parental involvement during transition to adult care for patients, parents experiencing feeling loss of stability and support surrounding the transition of their child's care, and significant nonmedical life events occurring for youths at the time of transition of care. Parents assuming supportive roles which change alongside their maturing child's needs were reported as being beneficial to young peoples' transition processes, while parents who hover over or micromanage their children during this time were found to hinder successful transitions. Ultimately, the majority of reviewed articles emphasized maintained parental involvement as having a net positive impact on adolescents' transitions to adult care. As such, practice and policies should be structured to engage parents throughout the transition process to best support their chronically ill children during this time of change.

## 1. Introduction

Chronic illnesses are conditions without cures [1], which have recurrent or continuous symptoms, though the timeline of what defines a chronic disease remains contested in the literature [2]. In recent years, many congenital chronic conditions—as well as chronic conditions diagnosed during childhood and adolescence—are associated with increasing rates of survival into adulthood [3]. Accordingly, there is growing demand to support young people with chronic diseases as they transition from paediatric to adult care settings. However, targeted research efforts to meet this demand are complicated by ambiguity surrounding the delineation of child (under 18 years old), adolescent (10-19 years), youth (15-24 years), and young people (10-24 years), the ages of which overlap in official international definitions [4].

These years of early adulthood are marked by continuing development, as well as environmental changes for youth with chronic diseases who require regular medical observation [5–7]. A key transition during this period involves a shift in care management. Decisions go from being made by parents, the patient, and healthcare providers based on the family-centred care (FCC) model generally utilized in paediatric settings to primarily the patient and their medical team based on the patient-centred care (PCC) model used in adult care [8, 9]. The challenges this transition poses were found to be ameliorated by an appropriate degree of parental involvement in a longitudinal cohort study from the United Kingdom [10]. Meaning, there is a balance of responsibility for the transitioning adolescent's care which is “dynamic and…continuously chang[ing]” between the parent and child [10]. How that balance can be struck and facilitated by medical care teams remains to be seen as parents are uncertain of how to play both a less active role in their child's care management, while continuing to provide them with support [11]. This change is further underscored by the fact that parents are undergoing their own transition during this time, from parenting a child with chronic illness to a young adult with said condition(s) [11]. While there are guidelines outlining the logistic steps involved in transitioning paediatric patients to adult care, which note the importance of family in patients' wellbeing during this time, little guidance is provided as to how family should be involved [12, 13].

When the transition process progresses to the point of the actual transfer to adult care occurring, young people with chronic illnesses are at increased risk for the development of care gaps [14]. Care gaps are defined as the difference between the provision of best available care and the care which is actually being received by patients [15]. In the case of adolescents' transfers to adult care, care gaps arise when patients either do not see their adult specialist providers at all or regularly enough as is indicated by the level of surveillance required to appropriately manage their condition(s) [14, 16]. This leads to decreases in patients' health maintenance and increases in adverse outcomes [14, 16].

As such, various transitional care programs have been developed and researched in attempts to support adolescents with chronic diseases before, during, and after this transfer period [17–19]. However, a 2022 scoping review notes that lacking on-going funding for transition programs in adult care settings and limited clinician knowledge surrounding the transition to adult care itself act as barriers to the optimal implementation of such supports [20]. Moreover, there is a general sparsity of research efforts in relation to young people with chronic illnesses once they have entered the adult system [21].

There also appears to be a lack of this research specifically investigating the role that parents could play to support their child through this transitional care process from a North American perspective. While exceptions do arise, in most Canadian settings, the transfer to adult care occurs at age 18 [13] and between the ages of 18 and 21 in the United States (US) [22]. However, patients' developmental states at their time of transfer vary between individuals [6]; that is to say, one eighteen-year-old may be at a point in their transition process where they are ready for the transfer to adult care, while another may not be. Therefore, there is a need to address requirements for successful transition to adult care based on adolescents' developmental stages, rather than chronological age [6]. Successful transition to adult care is being defined as “care that is continuous, coordinated, and adapted to each youth's development and maturity, while improving (or at least maintaining) disease control, patient satisfaction, quality of life, and social participation throughout young adulthood” [23].

Maintaining parental involvement in patients' care management may be a way to facilitate a successful transition to adult care. Colver et al. describe appropriate parental involvement to be when the young person and parent are both “satisfied” with the degree the parent remains engaged with their child's care management once they transition to adult services [10]. Examples of maintained parental involvement may range from parents attending clinic visits as silent participants to parents communicating with healthcare providers on behalf of their child [10]. Having young adults with chronic illnesses develop the skills necessary to independently manage the parts of their medical care that they can is a necessary component of successful transition [24]. However, as noted previously, patients will be at different stages of development as they transition to adult care, and therefore, they will have differing individualized needs in the process of cultivating such self-management skills [6]. Parental involvement in care and its known influence of “enhancing capacity to manage the [patient's] treatment regime” during childhood [25] may therefore be a useful tool to be maintained during the transition to adult care for emerging adults.

Whether and how continued engagement of parental support can serve to address the requirements for successful transition remains open as different studies have identified opposing findings regarding impacts of maintained parental involvement throughout this transition process [11, 26]. Accordingly, this scoping review addressed the following research question: “What is the effect of maintaining parental involvement in the care management of adolescents in early adulthood with chronic diseases during the transition from paediatric to adult care in North America?”

## 2. Materials and Methods

**Figure 1:**
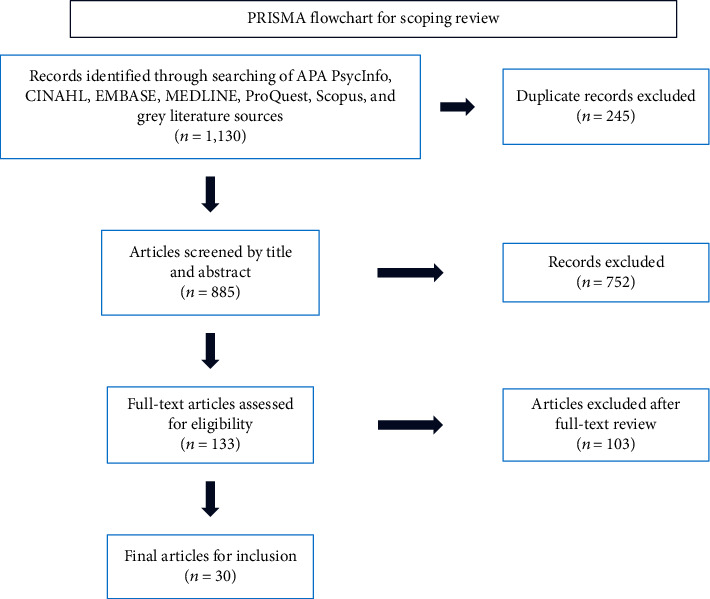
PRISMA flowchart for scoping review. This flowchart displays the process of elimination through which the final 30 articles included in this scoping review were selected.

This scoping review followed Arksey and O'Malley's [27] five-step framework as well as PRISMA-ScR guidelines [28] and includes the PRISMA flow chart (Figure 1). It was initially conducted from September 2020 to February 2021 as part of an independent Health Study research project (BB), with the searches rerun on March 3, 2022, to stay current. Articles were selected from 2010 onward to capture contemporary trends in care transitions. After establishing the research question, literature was collected through searches of electronic databases including APA PsycInfo, Cumulative Index to Nursing and Allied Health Literature (CINAHL), EMBASE, MEDLINE, ProQuest, Scopus, and advanced Google searches (see “Search Strings” in Supplemental Materials (available here)). Both scholarly and grey literature sources were reviewed, with search string development guided by an experienced reference librarian at the University of Toronto.

The following inclusion criteria were used to determine articles' eligibility for inclusion into this review: (1) published in English; (2) published between 2010 and 2020 (revised to 2010-2022); (3) research that was completed in Canada or the United States; (4) research that considered adolescents/youth with chronic conditions transitioning to adult care; (5) parents, families, or guardians being noted in the title or abstract; and (6) youth studied in considered research who did not have conditions impacting their cognitive development (e.g., neurodevelopmental disorders) nor did they have mental illness (e.g., eating disorders). Regarding the sixth inclusion criteria, these patient populations were excluded because their care needs may demand parents to remain involved in their care management, potentially in the capacity of substitute decision-makers (SDMs) [29]. The potential of parents having to play the role of SDMs would nullify arguments as to the appropriateness of differing levels of parental involvement in these young chronically ill patients' care within the adult setting. As such, articles that included said patient populations were excluded from this scoping review.

Deduplication and title and abstract screening were conducted by two independent reviewers (AB, BB) following the noted inclusion criteria with conflicts resolved in discussion with the team. From an initial 1,130 sources, 133 articles underwent full-text review. Thirty studies (see “Data Extraction Table” in Supplemental Materials) were ultimately included (Figure 1) [30–59]. An extraction table was constructed for these thirty studies and then reviewed by two team members (BB, CAK). Thematic content analysis [60] allowed for collation of common or particularly relevant findings into final themes which aligned with this scoping review's research question.

The populations of focus for the included thirty studies were as follows: eleven articles (37%) studied both adolescents and parents [32, 34, 36, 37, 44, 50, 51, 53, 55–57], eight (27%) studied adolescents only [31, 33, 35, 38, 40, 42, 54, 59], four (13%) studied adolescents, their parents, and their healthcare providers [39, 45, 47, 49], three (10%) studied only the healthcare providers of adolescents with chronic conditions [48, 52, 58], three included studies (13%) were review articles [30, 43, 46], and one article (3%) studied both adolescents and their healthcare providers [41]. Fifteen included studies (50%) used questionnaires/surveys as measures [31, 32, 34, 36–40, 42, 44, 50, 55–57, 59]. Fifteen (50%) used focus groups or interviews and their accompanying content analyses to collect data for their research [33, 35, 39, 41, 44, 45, 47–49, 51–56]. Six studies (20%) further complemented their use of questionnaires through reviewing participants' medical records for factors like clinic attendance, medication adherence, and a variety of medical tests [32, 36, 38, 40, 50, 57]. Only one study (3%) considering type 1 diabetes involved a biological sample as part of their study, which was a dried blood spot HbA1c test that participants could do at home and mail in [31].

## 3. Results

Data from included studies were charted into a Microsoft Excel (Redmond, United States) document (see “Data Extraction Table” in Supplemental Materials). That document was then used to collate findings and to identify themes across studies via thematic content analysis [60]. Most studies included were articles published in journals, with the exception of one doctoral dissertation [53]. Of the considered articles, 24 (80%) were of American origin [30–32, 34–42, 44, 48–51, 53–59]. Five studies (17%) were Canadian [33, 45–47, 52], and one was a review including both American and Canadian data [43].

Looking to the types of studies considered in this review, three (10%) [30, 43, 46] were reviews, four (13%) [34, 37, 42, 50] were cross-sectional studies, seven (23%) [31, 32, 36, 38, 40, 57, 59] were prospective cohort studies, and sixteen (53%) [33, 35, 39, 41, 44, 45, 47–49, 51–56, 58] were qualitative studies which utilized varying methods of content analysis (see “Data Extraction Table” in Supplemental Materials).

The specific effects of maintaining parental involvement throughout the transition were considered by 24 of the 30 (80%) included studies [30–36, 38–41, 44, 46–50, 52–56, 58, 59]. The various ways parents lose their sense of stability and support throughout transition were identified in ten studies (33%) [30, 37, 39, 44–46, 51, 53, 57, 58]. Another nine studies (30%) noted the implications of nonmedical life events during youths' times of transition to adult care [33, 35, 36, 40, 45, 48, 49, 55, 59].

### 3.1. Maintaining Parental Involvement

Twenty-four of the 30 (80%) included studies specifically considered the degree of parental involvement in chronically ill adolescents' care management, alongside the identified need for patients to become more independent in their care management when transitioning to adult care [30–36, 38–41, 44, 46–50, 52–56, 58, 59]. Different conclusions were drawn regarding whether developing said increased adolescent independence was benefited by the maintenance of parental involvement during transition or not.

#### 3.1.1. Maintained Parental Involvement as Having Positive Impacts on Successful Transition

**Figure 2:**
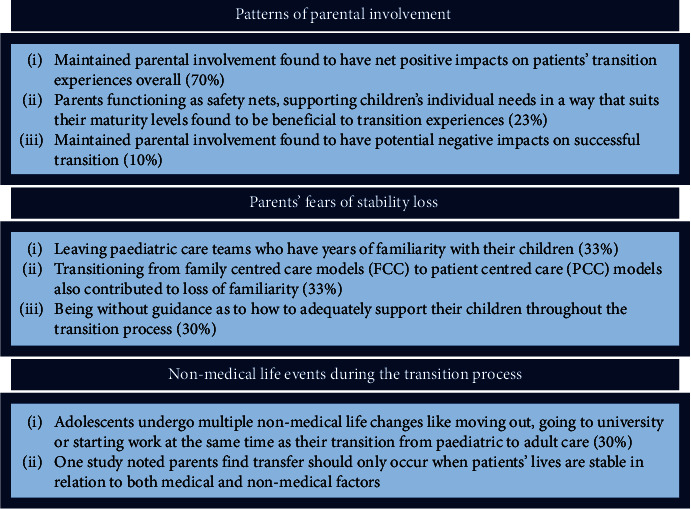
Themes and subtheme summary chart. This chart highlights the key themes and subthemes identified in the result portion of this scoping review.

Of those 24 studies, 21 (70%) viewed the continuation of parental involvement in young people with chronic illness' care management as having a net positive effect on patients' transitions to adult care (Figure 2) [30–33, 35, 36, 38, 40, 44, 46–50, 52–56, 58, 59]. Parental involvement was identified as playing an important role in adolescents' adherence to their medical regimens as dictated by their care needs during and after the transition to adult care [30–32, 40, 47, 52, 53, 55, 59].

Lack of parental involvement was also found to be problematic in two studies (7%) which identified that older adolescents, who were less monitored by their parents, were at higher risk for medication nonadherence [36, 38]. The value of parental involvement in care across multiple illnesses was further emphasized in three studies (10%), which found parents' comprehensive knowledge of medical regimens was positively correlated with their children having higher rates of clinic attendance [36, 38, 46].

#### 3.1.2. Parents as Safety Nets

Two of the 24 studies (8%) noted that parents function as “safety net[s]” [47, 58]. This was otherwise describable as backup supports for their children during transition to adult care by four other sources (13%) [41, 49, 50, 52]. This type of role was defined as parents remaining active participants in their child's medical care, however in a way that evolved to meet the needs of the young person as they became older. The specifics of what functioning as a “safety net” for adolescents entailed differed depending on the needs and independence level of the young person in question: the individualistic support needs of different patients during the transition process were noted across six (20%) studies [33, 48, 49, 52, 53, 59].

For some adolescents, maintained parental support involved parents attending appointments with them or making medical decisions on their behalf, well into adulthood (three sources, 10%) [33, 53, 59]. Other adolescents only looked to their parents for medical decision-making or care management support outside of appointments (six sources, 20%) [44, 47, 52, 53, 55, 59]. These individual-specific differing degrees of parental involvement were found to positively facilitate the transition process to adult care when they were predicated on respect for adolescents' wishes [48, 49].

#### 3.1.3. Nuances to the Positive Impacts of Maintained Parental Involvement

Six (20%) studies found that maintaining parental involvement in chronically ill youths' care management was a nuanced phenomenon in regard to successful transition, despite this involvement having a net positive effect [33, 35, 48, 53, 54, 58]. A noted nuanced aspect of maintained parental involvement included when parents and adolescents did not agree on treatment decisions when young people entered adult care [53]. Parents attending appointments against their child's wishes, monopolizing conversations with practitioners, and thus inhibiting the development of self-advocacy skills were found to have negative implications to successful transition [33, 35, 48]. “Helicopter parenting” as driven by fear of “let[ting] go” of their kids for concern of adverse health outcomes if they did was a prime example of how maintained parental involvement could hinder self-advocacy skill development [48]. Yet, despite these potential pitfalls, parents' involvement during transition to adult care was found to have net beneficial effects in 21 (70%) of the studies considering the degree of parental involvement during transition in this review [30–33, 35, 36, 38, 40, 44, 46–50, 52–56, 58, 59].

#### 3.1.4. Maintained Parental Involvement as Having Negative Impacts on Successful Transition

Three studies (10%) found that maintained parental engagement hindered successful transitions to adult care [34, 39, 41]. Two studies (7%) found that having high parental involvement in adolescents' care served as a barrier to chronically ill young people being able to adequately develop self-management skills [34, 39]. Adolescents with chronic conditions were found to defer to parents and play more passive roles regarding their treatment needs when parents played an active role in their care management [39, 41].

### 3.2. Parents and Loss of Stability and Support during and after Transition

Ten of the 30 (33%) included studies noted that parents experienced concern about their child leaving the paediatric environment or their paediatric care teams due to the latter's longstanding histories of treating these chronically ill youths [30, 37, 39, 44–46, 51, 53, 57, 58]. Alongside the apprehension of losing those relationships, changing models of care from FCC in paediatrics to PCC in adult facilities was another concern as noted in ten (33%) studies [30, 37, 39, 41, 43, 45, 51, 53, 54, 58].

This change in care models was identified as leading to a new set of expectations for patients and parents alike. Five studies (17%) found that chronically ill youth experience increased responsibility for their own care and their parents experience reduced levels of involvement in their children's medical care management and appointments [30, 39, 41, 45, 51]. Nine (30%) studies found that parents felt unsure or in need of guidance as to how they should be supporting their chronically ill children as such role changes occurred during the transition process [35, 36, 39, 42, 45–47, 53, 55]. Particularly as “comparable skills [to those involved with transition to adult care] are typically not demanded of [patients'] peers without a chronic illness until much further into adulthood” [58].

### 3.3. Nonmedical Life Events Occurring at the Time of Transition

Comparison to nonchronically ill youth allowed for the consideration of the nonmedical changes occurring during adolescence that chronically ill young people might likewise be facing, in nine of the 30 articles (30%) [33, 35, 36, 40, 45, 48, 49, 55, 59]. Nonmedical transitions might include going to postsecondary school, starting to work, or moving out, all of which involve new levels of independence from parents. These nonmedical life changes were noted by parents to make for an unideal time for transfer to adult care [39]. Such increased independence in chronically ill youths' nonmedical lives was also noted as potentially leading them to avoid treatment in attempts to save money [39], or not prioritizing their health in day-to-day life when critical events related to their condition (like major surgery) occurred in their infancy [43]. Both parents and children felt that transfer should only occur alongside “life stability” both in terms of “illness and life circumstances” [39]. Parents in one study suggested that transition should occur around age 25, an age they associated with increased stability and maturity compared to the age at which transitions currently occur [39].

## 4. Discussion

This scoping review considered the effects of maintaining parental involvement during the transition process from paediatric to adult care for young people with chronic diseases in North America. Importantly, the majority of reviewed studies found a net positive effect for continued parental involvement during the transition process, which has significant practice, policy, and research implications. This was similarly found in Swedish research which highlighted how parents could serve as valuable resources during the transition process in terms of their knowledge regarding the specifics of their child's care needs [61]. Such findings emphasizing the value of parental involvement during the transition to adult care for youth with chronic conditions are reflective of a shift away from seeing parents as “a barrier to young people's independence; a view that still pervades contemporary narratives.” [62].

This review also found parents to be concerned about loss of relationships with paediatric providers and uncertain as how best to support their children through the transition process. Such stressors and uncertainties were also noted in a Dutch study, an English scoping review, and an international systematic review [11, 63, 64]. These threats to stability were found to further compound the social changes adolescents underwent outside of their medical conditions like starting postsecondary education and moving away from home.

### 4.1. Practice Implications

Findings from this scoping review highlighted the individualized needs that different adolescents have in terms of parental support before, during, and after their transition to adult care [33, 35, 45, 48, 53, 54, 58]. PCC in adult settings expects care decisions to primarily be made between patients and practitioners [30, 39, 41, 45, 51]. However, practices should reflect that for some adolescent patients, PCC will demand continued active inclusion of parents in youths' medical decision-making processes.

Youth with chronic illnesses need to have access to appropriate preparatory tools to develop their self-management skills before the transfer to adult care occurs, for example, the development of individual and/or family-specific self-care management training plans [65]. They need adequate support from staff who can provide continuity of care throughout the transition process and help these young people navigate their novel adult care systems. When optimal versions of these supports and tools are in place, then the need for continued parental involvement into adult care may change. As highlighted by Doucet et al., some facilitators to the successful implementation of these programs include having a transition coordinator, having interdisciplinary teams to address both adolescents' medical and social needs, and having patient peer support groups [20]. Examples of existent services to facilitate transitions to adult care include SickKids' Resource Navigation Service (formerly the Good 2 Go Program) [66] and the Children's Hospital of Philadelphia's Adult Care and Transition Team (ACTT) [67].

### 4.2. Policy Implications

**Figure 3:**
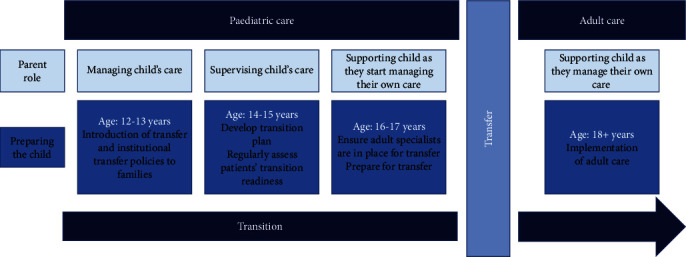
Age-focused transfer progress. This diagram displays a progression through the transition process if the stages of preparation are patient age-driven alone (without consideration of patients' individualized developmental needs).

Using age to determine when adolescents should be receiving paediatric compared to adult medical care may be necessary systemically to delineate the types of patients seen between these two settings as well as to generally guide practitioner and parent roles in patients' care during the adolescent years (Figure 3). However, regarding the latter point, some flexibility is necessary in interpreting such role changes and requirements. By considering differences in individual development and readiness for transition, youths' differing needs for support can be addressed and catered toward by their care providers, parents, and transition supports like those previously discussed. Such flexibility may be further fostered through the implementation of mandatory education about chronic diseases with childhood onsets for adult practitioners who may otherwise not be aware of the importance of parental involvement in these young adult patients' care management.

### 4.3. Research Implications

Further research with the primary objective of assessing the impacts of maintaining and optimizing parental involvement during and after chronically ill adolescents' transition to adult care is needed. Not a single included study was explicitly focused on assessing parental involvement in this way. Potential approaches for such future research may include prospective cohort studies assessing the degree of transition success for groups of young people with chronic illness in different transition programs which emphasize either maintained parental involvement or lack thereof as they move to adult care. Such studies should also assess the longer-term impacts of maintained parental involvement during transition on chronically ill youths' health management once they are situated in adult care. Adopting such an approach would help address the lack of longitudinal research available assessing transitions to adult care for this patient population. Additionally, it would be beneficial to establish specific factors of parental involvement which facilitate youth eventually developing skills like autonomous readiness for transition, which will be essential to their ability to manage their own healthcare as adults in the future.

It would also be useful to conduct comparative studies of different types of chronic conditions and or symptom severities to assess how such factors impact parents' roles and involvement in their child's care during transition. This may highlight shared needs for parental support across patient populations during transition, as well as condition or symptom severity-specific supports needed for particular patient groups. It may also be necessary to assess adult practitioners' understandings of adolescents' needs as they transition out of paediatric care. Analyses focusing on vulnerable subpopulations of chronically ill youth transitioning to adult care, like racialized patients or youths living in shelters, may be considered as well.

## 5. Limitations

Different studies used the term “adolescents” to describe various age categories. Some focused on youth as young as 12 years old, while others focused on patients in their late teens and early twenties. Resultantly, the transition process was assessed at very different points of patients' development across different studies. This potentially diluted noted themes for different age groups and their parents' transition concerns and experiences. As well, there is no standardized tool to assess transition success for patients or parents, and different questionnaires and interview structures were used across studies. This may have similarly limited comparability of articles. Only English language studies were considered for this review due to limited access to translators. As well, no bias assessment of the included research was conducted so as to include a breadth of studies in line with Arksey and O'Malley's framework [27]. Moreover, only North American studies were considered for this review. Though this allowed for potentially context-specific themes to be identified, lack of inclusion of international literature limits the comprehensiveness of identified findings.

## 6. Conclusions

Based on the themes identified across included articles in this scoping review, maintained parental involvement appears to have net positive impacts on young people with chronic illness' successful transitions to adult care. Patient-specific approaches to transition preparation and changes in parental involvement in youths' care were found to be facilitators of successful transition, rather than one-size-fits-all approaches. Thus, paediatric and adult healthcare providers should seek to work collaboratively alongside parents to support adolescents during this time of change. However, how parents remain involved in their adolescents' care is a nuanced phenomenon. Future research is needed to guide families, practitioners, and policy makers regarding how to best support young people with chronic illness as they transition to adult care.

## Supplementary Materials

Supplementary Materials Data Extraction Table: containing included research article information such as aims and objectives, design and methods, and outcome measures. Search Strings: a comprehensive list of the search strings used for each database searched in this scoping review.
