# Supplementary material for: Parental Involvement in the Transition from Paediatric to Adult Care for Youth with Chronic Illness: A Scoping Review of the North American Literature
Source: Int J Pediatr. 2023 Nov 24;2023:9392040. doi: 10.1155/2023/9392040 (PMC10691897; doi:10.1155/2023/9392040)
Supplement: Supplementary Materials — Data Extraction Table: containing included research article information such as aims and objectives, design and methods, and outcome measures. Search Strings: a comprehensive list of the search strings used for each database searched in this scoping review. [file 9392040.f1.pdf]

Article #	Article Information	Aims & Objectives	Design/Methods-Participants	Outcomes-Limitations
30	Abraham & Khan, Transition of care in inflammatory bowel disease,<br>Gastrology and Hepatology, USA, 2014	Overview of current practices, challenges, resources, and recommendations surrounding the transition to adult care for patients with inflammatory bowel disease (IBD).	Review	Parents experiencing a loss of stability during their child's transition to adult care.<br>Maintained parental support throughout the transition process as having a net positive impact on youths' transition to adult care.<br>Parental involvement during and after transition influencing youths' adherence to their medical care needs.<br>Parental worries surrounding changing models of care between paediatric and adult settings.
31	Baker et al., Structural model of patient-centered communication and diabetes management in early emerging adults at the transfer to adult care,<br>Journal of Behavioral Medicine, USA, 2019	To assess whether:<br>1) Young adults (18-25 years) with Type 1 diabetes who have high-quality relationships with mothers, have greater perceptions of patient centred care with their physicians<br>2) PCC is or is not associated with better adherence and glycemic control through diabetes related self efficacy.	Prospective cohort study<br>247 patients	Maintained parental support throughout the transition process as having a net positive impact on youths' transition to adult care.<br>Parental involvement during and after transition influencing youths' adherence to their medical care needs.
32	Bilhartz et al., Assessing allocation of responsibility for health management in pediatric liver transplant recipients,<br>Pediatric Transplantation, USA, 2015	Assessment of a new measure of allocation of responsibility in assessing health management abilities of pediatric liver transplant patients.	Prospective cohort study<br>48 patients<br>37 parents	Maintained parental support throughout the transition process as having a net positive impact on youths' transition to adult care.<br>Parental involvement during and after transition influencing youths' adherence to their medical care needs.

33	Catena et al., "Not such a kid thing anymore": Young adults' perspectives on transfer from paediatric to adult cardiology care, Child: Care, Health and Development, Canada, 2018	Examining the perspectices of young adults with congenital heart disease (CHD) about their experiences having transferred to adult congenital heart disease (ACHD) care as compared to those who continue to receive care in paediatric centres.	Thematic content analysis<br>21 patients	Youth experiencing significant non-medical life changes or events during the time of transition to adult care.<br>Maintained parental support throughout the transition process as having a net positive impact on youths' transition to adult care.<br>Nuances to the positive impacts of maintained parental involvement in transition.
34	Chandra et al., Growing up: Not an easy transition -- Perspectives of patients and parents regarding transfer from pediatric liver transplant center to adult care, International Journal of Hepatology, USA, 2015	To assess the difficulties faced by transplant patients during their transition to adult care prior to the creation of a formal transition program as well as to collect suggestions regarding the transition process.	Cross-sectional study<br>12 patients<br>19 parents	Maintained parental involvement during the transition process as having negative implications for youths' successful transition.
35	Frederick et al., Preparing childhood cancer survivors for transition to adult care: The young adult perspective, Pediatric Blood & Cancer, USA, 2017	Assess the educational, information, and support needs of childhood cancer survivors (CCS) as they transition from paediatric to adult care.	Thematic content analysis of focus group<br>16 patients	Youth experiencing significant non-medical life changes or events during the time of transition to adult care.<br>Maintained parental support throughout the transition process as having a net positive impact on youths' transition to adult care.<br>Nuances to the positive impacts of maintained parental involvement in transition.<br>Parents unsure how to support their child through transition.

36	Fredericks et al., Assessment of transition readiness skills and adherence in pediatric liver transplant recipients, Pediatric Transplantation, USA, 2010	Investigating pediatric liver transplant recipients' transition readiness, treatment adherence, and health outcomes using a screening tool.	Prospective cohort study<br>71 patients<br>58 parents	Youth experiencing significant non-medical life changes or events during the time of transition to adult care.<br>Maintained parental support throughout the transition process as having a net positive impact on youths' transition to adult care.<br>Parents unsure how to support their child through transition.
37	Fredericks et al., Transition of pediatric liver transplant recipients to adult care: Patient and parent perspectives, Pediatric Transplantation, USA, 2011	To assess young adult liver transplant recipients and their parents' perceptions and attitudes towards the transition to adult care.	Cross-sectional study<br>46 patients<br>31 parents	Parents experiencing a loss of stability during their child's transition to adult care.<br>Parental worries surrounding changing models of care between paediatric and adult settings.
38	Fredericks et al., Quality improvement targeting adherence during the transition from pediatric to adult liver transplant clinic, Journal of Clinical Psychology in Medical Settings, USA, 2015	Investigating a quality improvement project for pediatric liver transplant recipients aimed at improving health outcomes after transfer to adult care occurs.	Prospective cohort study<br>45 patients	Maintained parental support throughout the transition process as having a net positive impact on youths' transition to adult care.<br>Parental involvement during and after transition influencing youths' adherence to their medical care needs.

39	Gray et al., Concerns, barriers, and recommendations to improve transition from pediatric to adult IBD care: Perspectives of patients, parents, and health professionals, Inflammatory Bowel Diseases, USA, 2015	Focus groups conducted to assess adolescents', their healthcare professionals', and their parents' worries and perceived needs regarding the transition to adult care.	Direct content analysis of focus group<br>15 patients<br>16 parents<br>13 healthcare professionals	Maintained parental involvement during the transition process as having negative implications for youths' successful transition.<br>Parents experiencing a loss of stability during their child's transition to adult care.<br>Parents unsure how to support their child through transition.<br>Parental worries surrounding changing models of care between paediatric and adult settings.
40	Helgeson et al., Characterizing the transition from paediatric to adult care among emerging adults with Type 1 diabetes, Diabetic Medicine, USA, 2013	Describes the transition to adult care for youth with Type 1 diabetes (T1D). Assesses the connection between self care and glycaemic control as well as if parent relationship variables were associated with patients seeing adult or paediatric physicians for their care.	Prospective cohort study<br>118 patients	Youth experiencing significant non-medical life changes or events during the time of transition to adult care.<br>Maintained parental support throughout the transition process as having a net positive impact on youths' transition to adult care.<br>Parental involvement during and after transition influencing youths' adherence to their medical care needs.
41	Huang et al., Transition to adult care: Systematic assessment of adolescents with chronic illnesses and their medical teams, The Journal of Pediatrics, USA, 2011	Considers the transition to adult care from the perspective of adolescents with chronic diseases as well as from paediatric and adult healthcare providers in terms facilitators and barriers to transition and how Web-based technologies can support this transition.	Content and narrative analysis of focus group interviews<br>10 patients<br>24 healthcare professionals	Maintained parental involvement during the transition process as having negative implications for youths' successful transition.<br>Parental worries surrounding changing models of care between paediatric and adult settings.

42	Johnson et al., The relationship of transition readiness, self-efficacy, and adherence to preferred health learning method by youths with chronic conditions, The Journal of Pediatric Nursing, USA, 2015	To assess whether young people who prefer collecting health information about their conditions from their healthcare providers are more likely to have higher transition readiness scores than those who prefer collecting said information from their family, the internet, or peers with the same condition.	Cross-sectional study<br>160 patients	Parents unsure how to support their child through transition.
43	Kovacs & McCrindle., So hard to say goodbye: transition from paediatric to adult cardiology care, Nature Reviews Cardiology, USA & Canada, 2013	Review of existent information on the transition process for patients with congential/paediatric-onset cardiovascular conditions considering patient, adult/paediatric provider, and parent stakeholders.	Review	Parental worries surrounding changing models of care between paediatric and adult settings.
44	Lee et al., The Development of Health Self-Management Among Adolescents With Chronic Conditions: An Application of Self-Determination Theory, Journal of Adolescent Health, USA, 2021	Investigating the process through which self-management skills are developed for adolescents' with chronic conditions through use of interviews with patients and their parents and the application of Self Determination Theory.	Direct content analysis of interviews<br>23 sets of patients and parents	Parents experiencing a loss of stability during their child's transition to adult care.<br>Maintained parental support throughout the transition process as having a net positive impact on youths' transition to adult care.<br>Parents functioning as "safety nets" (or equivalent) for youth during transition to adult care.

45	Lindsay et al., Enablers and barriers of men with Duchenne muscular dystrophy transitioning from an adult clinic within a pediatric hospital, Disability and Health Journal, Canada, 2017	Identifies barriers and facilitators for patients, care providers, and parents in the successful transition of young men with Duchenne muscular dystrophy (DMD) from paediatric to adult settings of care.	Thematic content analysis of interviews<br>4 patients<br>5 parents<br>7 healthcare professionals	Parents experiencing a loss of stability during their child's transition to adult care.<br>Youth experiencing significant non-medical life changes or events during the time of transition to adult care.<br>Parents unsure how to support their child through transition.<br>Parental worries surrounding changing models of care between paediatric and adult settings.
46	Mackie et al., Transition and transfer from pediatric to adult congenital heart disease in Canada: Call for strategic implementation, Canadian Journal of Cardiology, Canada, 2019	To summarize current literature regarding the transition process, from multiple different stakeholders' perspectives so as to propose implementation recommendations to improve said process.	Review	Parents experiencing a loss of stability during their child's transition to adult care.<br>Maintained parental support throughout the transition process as having a net positive impact on youths' transition to adult care.<br>Parents unsure how to support their child through transition.
47	Nguyen et al., You never transition alone! Exploring the experiences of youth with chronic health conditions, parents and healthcare providers on self-management, Child: Care, Health and Development, Canada, 2016	Investigating adolescents with chronic health conditions, their health providers' and their parents' perspectives towards self-management during the transition to adult care.	Interpretive phenomenology of interviews<br>11 patients<br>7 parents<br>7 healthcare professionals	Maintained parental support throughout the transition process as having a net positive impact on youths' transition to adult care.<br>Parental involvement during and after transition influencing youths' adherence to their medical care needs.<br>Parents functioning as "safety nets" (or equivalent) for youth during transition to adult care.<br>Parents unsure how to support their child through transition.

48	Paine et al., Barriers and facilitators to successful transition from pediatric to adult inflammatory bowel disease care from the perspectives of providers, Inflammatory Bowel Diseases, USA, 2014	Identification of key outcomes to assess success of transition to adult care for adolescents with inflammatory bowel disease (IBD) as well as noting barriers and enablers to transition for this population.	Thematic content analysis of interviews<br>12 healthcare professionals	Youth experiencing significant non-medical life changes or events during the time of transition to adult care.<br>Maintained parental support throughout the transition process as having a net positive impact on youths' transition to adult care.<br>Nuances to the positive impacts of maintained parental involvement in transition.
49	Pierce et al., Health care transition for young adults with Type 1 diabetes: Stakeholder engagement for defining optimal outcomes, Journal of Pediatric Psychology, USA, 2017	Engagement of adolescents with Type 1 diabetes (T1D), parents, paediatric, and adult care providers, and other experts in interviews to guide the development of multiple outcomes to be used for the assessment of successful/unsuccessful health care transitions (HCTs).	Direct content analysis of interviews<br>10 patients<br>9 parents<br>18 healthcare professionals	Youth experiencing significant non-medical life changes or events during the time of transition to adult care.<br>Maintained parental support throughout the transition process as having a net positive impact on youths' transition to adult care.
50	Polfuss et al., Family perspectives of components of a diabetes transition program, Journal of Pediatric Nursing, USA, 2015	Assessment of adolescents with Type 1 diabetes (T1D) and their parents' perspectives of self-efficacy, self-management, knoweldge of diabetes, knowledge and skills needed for the transition to adult care.	Cross-sectional study<br>45 patient-parent dyads	Maintained parental support throughout the transition process as having a net positive impact on youths' transition to adult care.

51	Psihogios et al., Preferences for cancer survivorship care among adolescents and young adults who experienced healthcare transitions and their parents, Journal of Cancer Survivorship, USA, 2019	Investigating patients' and parents' perceptions towards the value, availability, and goals included in long-term-follow-up care for childhood cancer survivors (CCS).	Direct content analysis of focus group interviews<br>14 patients<br>15 parents	Parents experiencing a loss of stability during their child's transition to adult care.<br>Parental worries surrounding changing models of care between paediatric and adult settings.
52	Schraeder et al., Perspectives from primary health care providers on their roles for supporting adolescents and young adults transitioning from pediatric services, BMC Family Practice, Canada, 2020	To understand family physicians' and other primary health care providers' perspectives towards how their involvement in chronically ill young peoples' transition to adult care either benefits or hinders the process.	Thematic content analysis of interviews<br>18 healthcare professionals	Maintained parental support throughout the transition process as having a net positive impact on youths' transition to adult care.<br>Parental involvement during and after transition influencing youths' adherence to their medical care needs.<br>Parents functioning as "safety nets" (or equivalent) for youth during transition to adult care.
53	Sibayan., Family relational experiences during major transitions with a chronic illness, [Doctoral Thesis - Walden University], USA, 2018	Investigation of the lived experiences of young adults with chronic conditions and their parents during the transition from paediatric to adult care.	Thematic content analysis of interviews<br>6 patients<br>8 parents	Maintained parental support throughout the transition process as having a net positive impact on youths' transition to adult care.<br>Parents experiencing a loss of stability during their child's transition to adult care.<br>Parental involvement during and after transition influencing youths' adherence to their medical care needs.<br>Parents functioning as "safety nets" (or equivalent) for youth during transition to adult care.<br>Parents unsure how to support their child through transition.<br>Nuances to the positive impacts of maintained parental involvement in transition.<br>Parental worries surrounding changing models of care between paediatric and adult settings.

54	Simms et al., Health communication experiences of emerging adults with Type 1 diabetes, Clinical Practice in Pediatric Psychology, USA, 2017	Creation of a conceptual model for the care of emerging adults with T1D through considering these patients' health-communication experiences with healthcare providers.	Conventional content analysis of interviews<br>20 patients	Maintained parental support throughout the transition process as having a net positive impact on youths' transition to adult care.<br>Nuances to the positive impacts of maintained parental involvement in transition.<br>Parental worries surrounding changing models of care between paediatric and adult settings.
55	Sliwinski et al., Transitioning from pediatric to adult health care with familial hypercholesterolemia: Listening to young adult and parent voices, Journal of Clinical Lipidology, USA, 2017	Idenitification of difficulties in transition for adolescents with familial hypercholesterolemia (FH) and their parents in relation to two tasks surrounding responsibility and self management of care.	Modified grounded theory framework analysis of interviews<br>12 patients<br>12 parents	Youth experiencing significant non-medical life changes or events during the time of transition to adult care.<br>Maintained parental support throughout the transition process as having a net positive impact on youths' transition to adult care.<br>Parental involvement during and after transition influencing youths' adherence to their medical care needs.<br>Parents functioning as "safety nets" (or equivalent) for youth during transition to adult care.<br>Parents unsure how to support their child through transition.
56	Speller-Brown et al., Measuring transition readiness: A correlational study of perceptions of parent and adolescents and young adults with sickle cell disease, Journal of Pediatric Nursing, USA, 2015	Identification of perceptions of transition readiness in sickle cell disease (SCD) patients and their parents through consideration of patient age, responsibility for own care, and parental involvement in care management.	Descriptive correlational study design<br>60 patient-parent dyads	Maintained parental support throughout the transition process as having a net positive impact on youths' transition to adult care.

57	Stephany et al., Transition of urologic patients from pediatric to adult care: A preliminary assessment of readiness in Spina Bifida patients, Urology, USA, 2015	Investigation of spina bifida patients' and parents' awareness and readiness for transition to adult care with focus on factors associated with successful transitions from paediatric multidisciplinary teams to adult urological care.	Prospective cohort study<br>15 patients<br>18 parents	Parents experiencing a loss of stability during their child's transition to adult care.
58	Stollon et al., Transitioning adolescents and young adults with sickle cell disease from pediatric to adult healthcare: Provider perspectives, Journal of Pediatric Hematology/Oncology, USA, 2015	Investigating potential metrics for successful transition, as well as facilitators/barriers to transition through use of the SMART model framework as applied to young people with sickle cell disease (SCD).	Thematic content analysis<br>13 healthcare professionals	Parents experiencing a loss of stability during their child's transition to adult care.<br>Maintained parental support throughout the transition process as having a net positive impact on youths' transition to adult care.<br>Nuances to the positive impacts of maintained parental involvement in transition.<br>Parents functioning as "safety nets" (or equivalent) for youth during transition to adult care.<br>Parental worries surrounding changing models of care between paediatric and adult settings.
59	Szalda et al., Associates of engagement in adult-oriented follow-up care for childhood cancer survivors, Journal of Adolescent Health, USA, 2017	Assessing theoretically rooted factors of engagement in the adult care setting as applied to childhood cancer survivors (CCS) as well as if transition readiness measures are associated with care engagement after transfer to adult settings.	Prospective cohort study<br>80 patients	Youth experiencing significant non-medical life changes or events during the time of transition to adult care.<br>Maintained parental support throughout the transition process as having a net positive impact on youths' transition to adult care.<br>Parental involvement during and after transition influencing youths' adherence to their medical care needs.

Supplemental Materials: Search Strings

APA PsycInfo:
Limited to 2010-2020 in English October 29th, 2020 **Results: 85**
Limited to 2020-2022 in English, March 3rd, 2022 **Results: 9**

1. Adolescent development/
2. (Transition* to adult care). Tw
3. (transfer* to adult care). Tw
4. (mov* to adult care). Tw
5. (leav* pediatric care). Tw
6. (transfer* from pediatric to adult care). Tw
7. (care handover). Tw
8. 1 OR 2 OR 3 OR 4 OR 5 OR 6 OR 7
9. Exp Parent Child Relations/
10. (parent*).tw
11. (parent* involv*). Tw
12. (parent* inclu*). Tw
13. (maintain* parent* involv*). Tw
14. (parent* role*). Tw
15. 9 OR 10 OR 11 OR 12 OR 13 OR 14
16. Exp Chronic Illness
17. chronic disease*
18. medical complexit*
19. multi-morbidit*
20. congenital condition*
21. 17 OR 18 OR 19 OR 20
22. 8 AND 15 AND 21

CINAHL Plus:
Limited to 2010-2019 (no 2020 option), October 29th, 2020 **Results: 13**
Limited to 2020-2022, March 3rd, 2022 **Results: 4**

1. (MH "Adolescent Development") OR (MH "Transition to Adulthood")
2. "Transition* to adult care"
3. "transfer* to adult care"
4. "leav* pediatric care"
5. 1 OR 2 OR 3 OR 4
6. (MH "Chronic disease")
7. "congenital condition"
8. "multiple morbidities"
9. 6 OR 7 OR 8
10. (MH "Parent-Child Relations+")
11. (MH "Parents") OR (MH "Parents, Disabled") OR (MH "Parents of Disabled Children")
12. (MH "Parental Role")

13. “parent inclusion”
14. “parental involvement”
15. 10 OR 11 OR 12 OR 13 OR 14
16. 5 AND 9 AND 15

EMBASE:
Limited to 2010-2020 in English, October 29th, 2020 **Results: 65**
Limited to 2020-2022 in English, March 3rd, 2022 **Results: 14**

1. Exp transition to adult care/
2. transition* to adult care
3. care handover
4. transfer from pediatric to adult care
5. mov* to adult care
6. leav* pediatric care
7. 1 OR 2 OR 3 OR 4 OR 5 OR 6
8. Exp child parent relations/
9. (parent*). tw
10. (parent* involv*). tw
11. (parent* role). Tw
12. (parent* participat*). tw
13. (parent* inclu*). tw
14. (continu* parent* involve*).tw
15. 8 OR 9 OR 10 OR 11 OR 12 OR 13 OR 14
16. Chronic disease/
17. chronic* illness
18. multi-morbidit*
19. congenital condition*
20. medical complexit*
21. 16 OR 17 OR 18 OR 19 OR 20
22. 7 AND 15 OR 21

MEDLINE:
Limited to 2010-2020 in English, October 29th, 2020 **Results: 52**
Limited to 2020-2022 in English, March 3rd, 2022 **Results: 10**

1. Transition to adult care/
2. transition* to adult care
3. transfer* to adult care
4. enter* adult care
5. transfer from pediatric to adult care
6. mov* to adult care
7. 1 OR 2 OR 3 OR 4 OR 5 OR 6
8. exp Parent-child Relations/
9. (parent*). tw
10. (parent* involv*). Tw
11. (parent* role*). Tw
12. (parent* inclu*). Tw
13. (parent* support*). Tw
14. (maintain* parent* involv*). Tw
15. 9 OR 10 OR 11 OR 12 OR 13 OR 14
16. exp Chronic Disease/
17. chronic illness*
18. multi-morbit*
19. congenital condition*
20. medical complexit*
21. medical dependenc*
22. child* with medical complexit*
23. 16 OR 17 OR 18 OR 19 OR 20 OR 21 OR 22
24. 7 AND 15 AND 23

ProQuest:
Limited 2010-2020 in English, October 29th, 2020 **Results: 155, 978**
Additional limits: scholarly journals, humans, 2010-2020
Note: Considered **1500 articles** of those results
-Stopped after page 15 (100 results per page) as less than 15% of the articles were remotely close to being relevant to the study between page 12 and 15
Rerun with noted limitations for 2020-2022 in English, March 3rd, 2022 with **no change**

1. Noft(transition to adult care)
2. Noft(transfer* to adult care)
3. Noft(transition from pediatric to adult care)
4. 1 OR 2 OR 3
5. Ab(parent-child relationship)
6. Ab(parent)
7. Ab(parent roles)
8. Ab(parents involvement)
9. Ab(parents inclusion)
10. 5 OR 6 OR 7 OR 8 OR 9
11. Noft(Chronic diseases)
12. Noft (Chronic illnesses)
13. 11 OR 12
14. 4 AND 10 AND 13

Scopus:

Limited 2010-2020 in English, October 29th, 2020 **Results: 57**
Limited 2020-2022 in English, March 3rd, 2022 **Results: 0**

1. "Adolescent transition to adult care"
2. "Adolescent transfer* to adult care"
3. "Adolescent mov* to adult care"
4. 1 OR 2 OR 3
5. "parent-child relation*"
6. "parent*"
7. "parent* role"
8. "parent* involve*"
9. "parent* participation"
10. 5 OR 6 OR 7 OR 8 OR 9
11. chronic disease*
12. chronic illness*
13. multi* morbit*
14. congenital condition*
15. 11 OR 12 OR 13 OR 14
16. 4 AND 10 AND 15

Grey Literature

ProQuest Dissertations & Theses Global, October 29th, 2020 **Results: 26**
Limited to full text, 2010-2020
ProQuest Dissertations & Theses Global, March 3rd, 2022 **Results: 0**
Limitted to full text, 2020-2022

Selected: 4
1. Noft(pediatric transition to adult care)
2. Noft(parents)

<u>Google: October 29th, 2020</u>
Search 1: "British Columbia Children's Hospital transition to adult care"
Results: 5,770,000
→ First page considered after which results considered other provinces and information sources besides specific search
→ Selected: 3

Search 2: "Alberta Children's Hospital transition to adult care"
Results: 3,580,000
→ First page of results considered
→ Selected: 1

Search 3: Children's Healthcare Canada transition to adult care
Results: 44,400,000
→ First page considered
→ Selected: 1
